# A genetic risk score combining 32 SNPs is associated with body mass index and improves obesity prediction in people with major depressive disorder

**DOI:** 10.1186/s12916-015-0334-3

**Published:** 2015-04-17

**Authors:** Chi-Fa Hung, Gerome Breen, Darina Czamara, Tanguy Corre, Christiane Wolf, Stefan Kloiber, Sven Bergmann, Nick Craddock, Michael Gill, Florian Holsboer, Lisa Jones, Ian Jones, Ania Korszun, Zoltan Kutalik, Susanne Lucae, Wolfgang Maier, Ole Mors, Michael J Owen, John Rice, Marcella Rietschel, Rudolf Uher, Peter Vollenweider, Gerard Waeber, Ian W Craig, Anne E Farmer, Cathryn M Lewis, Bertram Müller-Myhsok, Martin Preisig, Peter McGuffin, Margarita Rivera

**Affiliations:** MRC SGDP Centre, Institute of Psychiatry, Psychology & Neuroscience, King’s College London, Box PO82, De Crespigny Park, Denmark Hill, London, SE5 8AF UK; Department of Psychiatry, Kaohsiung Chang Gung Memorial Hospital and Chang Gung University College of Medicine, Kaohsiung, 833 Taiwan; National Institute for Health Research Biomedical Research Centre for Mental Health at the Maudsley and Institute of Psychiatry, King’s College London, London, UK; Max-Planck-Institute of Psychiatry, Kraepelinstraße 2, 80804 Munich, Germany; Institute of Social and Preventive Medicine (IUMSP), Centre Hospitalier, Universitaire Vaudois (CHUV), 1010, Lausanne, Switzerland; Swiss Institute of Bioinformatics, Lausanne, 1015 Switzerland; MRC Centre for Neuropsychiatric Genetics and Genomics, Cardiff University, Hadyn Ellis Building, Maindy Road, Cardiff, CF24 4HQ UK; Department of Psychiatry, Trinity Centre for Health Sciences, Dublin 8, Ireland; Department of Psychiatry, School of Clinical and Experimental Medicine, University of Birmingham, Birmingham, B15 2TT UK; Barts and The London School of Medicine and Dentistry, Queen Mary’s University of London, London, E1 2AD UK; Department of Psychiatry, University of Bonn, 53127 Bonn, Germany; Research Department P, Aarhus University Hospital, Skovagervej 2, DK-8240 Risskov, Denmark; MRC Centre for Neuropsychiatric Genetics and Genomics, Department of Psychological Medicine and Neurology, School of Medicine, Cardiff University, Henry Wellcome Building, Heath Park, Cardiff, CF14 4XN UK; Department of Psychiatry, Washington University School of Medicine, St Louis, MO 63130 USA; Central Institute of Mental Health, 68159 Mannheim, Germany; Department of Psychiatry, Dalhousie University, Halifax, Nova Scotia, NS B3H 3J5 Canada; Division of Internal Medicine, CHUV, Rue du Bugnon 21, 1011, Lausanne, Switzerland; Department of Medical and Molecular Genetics, School of Medicine, King’s College London, 8th Floor, Tower Wing, Guys Hospital, London, SE1 9RT UK; Department of Psychiatry, Lausanne University Hospital, 1008 Prilly-Lausanne, Switzerland; CIBERSAM-University of Granada and Institute of Neurosciences Federico Olóriz, Centro de Investigación Biomédica, University of Granada, Avda del Conocimiento s/n, 18100 Armilla, Granada, Spain; Instituto de Investigación Biosanitaria ibs.GRANADA, Hospitales Universitarios de Granada/Universidad de Granada, 18012, C/ Dr. Azpitarte, 4, 18012, Granada, Spain

**Keywords:** Body mass index, Genetic risk score, Major depressive disorder, Obesity

## Abstract

**Background:**

Obesity is strongly associated with major depressive disorder (MDD) and various other diseases. Genome-wide association studies have identified multiple risk loci robustly associated with body mass index (BMI). In this study, we aimed to investigate whether a genetic risk score (GRS) combining multiple BMI risk loci might have utility in prediction of obesity in patients with MDD.

**Methods:**

Linear and logistic regression models were conducted to predict BMI and obesity, respectively, in three independent large case–control studies of major depression (Radiant, GSK-Munich, PsyCoLaus). The analyses were first performed in the whole sample and then separately in depressed cases and controls. An unweighted GRS was calculated by summation of the number of risk alleles. A weighted GRS was calculated as the sum of risk alleles at each locus multiplied by their effect sizes. Receiver operating characteristic (ROC) analysis was used to compare the discriminatory ability of predictors of obesity.

**Results:**

In the discovery phase, a total of 2,521 participants (1,895 depressed patients and 626 controls) were included from the Radiant study. Both unweighted and weighted GRS were highly associated with BMI (*P* <0.001) but explained only a modest amount of variance. Adding ‘traditional’ risk factors to GRS significantly improved the predictive ability with the area under the curve (AUC) in the ROC analysis, increasing from 0.58 to 0.66 (95% CI, 0.62–0.68; χ^2^ = 27.68; *P* <0.0001). Although there was no formal evidence of interaction between depression status and GRS, there was further improvement in AUC in the ROC analysis when depression status was added to the model (AUC = 0.71; 95% CI, 0.68–0.73; χ^2^ = 28.64; *P* <0.0001). We further found that the GRS accounted for more variance of BMI in depressed patients than in healthy controls. Again, GRS discriminated obesity better in depressed patients compared to healthy controls. We later replicated these analyses in two independent samples (GSK-Munich and PsyCoLaus) and found similar results.

**Conclusions:**

A GRS proved to be a highly significant predictor of obesity in people with MDD but accounted for only modest amount of variance. Nevertheless, as more risk loci are identified, combining a GRS approach with information on non-genetic risk factors could become a useful strategy in identifying MDD patients at higher risk of developing obesity.

**Electronic supplementary material:**

The online version of this article (doi:10.1186/s12916-015-0334-3) contains supplementary material, which is available to authorized users.

## Background

Obesity is a serious public health problem associated with an increased risk of various chronic diseases such as hypertension, diabetes, and cardiovascular disease [1]. It is estimated that over one-third of adults in the US are obese, whereas another one-third are overweight [2]. Moreover, the prevalence rate of obesity or overweight in most countries has been rising steadily over the past decades, resulting in a huge health burden [3]. There is also evidence that people with major depressive disorder (MDD) are more likely to be overweight or obese compared to psychiatrically-healthy controls [4], particularly in individuals with atypical depression, in whom increased appetite and weight gain are more prevalent. In addition, depressed people have a higher risk for various medical diseases and most of them are obesity-related. A recent meta-analysis further suggested the bi-directional relationship between obesity and MDD [5]. Given the high prevalence rate of both obesity and MDD, understanding the nature of their relationship is a pressing clinical problem.

Dietary factors and a lack of exercise as well as genetic factors contribute to the development of obesity. Twin and family studies have suggested the heritability of body mass index (BMI) to be between 0.4 and 0.7 [6]. The advance of genome-wide association studies (GWAS) has successfully identified multiple polymorphisms associated with the risk of obesity and higher BMI [7-9]. Among them, the fat mass and obesity associated (*FTO*) gene was consistently and reliably replicated in different studies. Our team has found that several polymorphisms in the *FTO* gene, the locus conferring the highest genetic risk contribution to obesity, are associated with increased BMI in people with MDD. A disease history of depression further moderates the effect of *FTO* on BMI [10]. However, each risk variant only confers a modest effect on the risk, resulting in a limited ability for obesity prediction by applying single variants. It has been suggested that combining multiple loci into a genetic risk score (GRS) might improve prediction of obesity. Although several studies have examined the joint genetic effect using different numbers of genetic variants to discriminate obesity in the general population [11-13], no study, to date, has investigated the combined genetic effects on obesity in people with MDD.

In this study, we aimed to investigate whether a GRS incorporating a number of well-defined common single nucleotide polymorphisms (SNPs) might have utility in prediction of obesity in patients with MDD.

## Methods

### Subjects and phenotypes

#### *Discovery phase***–***Radiant study*

A total of 3,244 participants (2,434 depressed patients and 810 healthy controls) were recruited from the Radiant study, which included the Depression Network (DeNT) study [14], the Depression Case–Control (DeCC) study [15], and the Genome-Based Therapeutic Drugs for Depression (GENDEP) study [16]. The DeNT study is a family study which recruited sibling pairs affected with recurrent unipolar depression from eight clinical sites across Europe and one in the USA. Only one proband from each family was recruited in our analysis. The DeCC study is a case–control study which recruited unrelated patients from three sites in the UK. All participants in the DeNT and DeCC studies experienced two or more episodes of major depression of at least moderate severity. The GENDEP study recruited individuals with at least one episode of depression of at least moderate severity from nine European centres. People who had ever fulfilled criteria of intravenous drug dependence, substance-induced mood disorder, schizophrenia, or bipolar disorder were excluded. The diagnosis of MDD was ascertained using the Schedules for Clinical Assessment in Neuropsychiatry (SCAN) [17] interview in all three studies. The controls were screened for lifetime absence of any psychiatric disorder using a modified version of the Past History Schedule [18]. Participants were excluded if they, or a first-degree relative, ever fulfilled the criteria for depression, bipolar disorder, or schizophrenia.

Self-reported weight and height were obtained during the SCAN interview for the individuals with depression and during telephone interview for controls. BMI was defined as weight in kilograms divided by height in meters squared. Obesity was defined as BMI ≥30 and normal weight was defined as BMI between 18.5 and 25. The reliability of self-report of height and weight was assessed in the GENDEP dataset (n = 811) where we also had measured height and weight. The correlations for measured versus self-reported height, weight, and BMI were 0.97, 0.95, and 0.95, respectively.

All participants were of white European ancestry. Approval was obtained from the local research ethics committees/institutional research boards of all of the participating sites. The full list of ethics committees can be seen in Additional file 1.

#### Replication phase – GSK-Munich study

Overall, 1,679 participants (822 cases and 857 controls) were recruited at the Max-Planck Institute of Psychiatry in Munich, Germany, and at two psychiatric hospitals in the Munich area (BKH Augsburg and Klinikum Ingolstadt). The same inclusion and exclusion criteria were applied in this study as the Radiant study. Patients had to fulfil the diagnosis of recurrent major depressive disorder of moderate or severe intensity using the SCAN interview. Controls were selected randomly from a Munich-based community and were screened for the presence of anxiety or mood disorders using the Composite International Diagnostic Screener (German version) [19]. Only individuals without mood and anxiety disorders were collected as controls. This study has been described in more detail elsewhere [20]. Anthropometric measures for patients and controls were taken at the Max Planck Institute and associated studies sites by trained technicians and study nurses [20].

This study was approved by the Ethics Committee of the Ludwig Maximilian University, Munich, Germany and written informed consent was obtained from all participants.

#### PsyCoLaus study

A total of 2,993 participants (1,296 cases and 1,697 controls) were recruited from a psychiatric sub-study (PsyCoLaus) of a community survey (CoLaus) carried out in Lausanne, Switzerland. A DSM-IV diagnosis of MDD was ascertained using the Diagnostic Interview for Genetics Studies [21]. The control subjects never fulfilled criteria for MDD. The PsyCoLaus study has been described in more detail elsewhere [22]. Weight and height were measured at the outpatient clinic at the Centre Hospitalier Universitaire Vaudois [23].

The Ethics committee of the Faculty of Biology and Medicine of the University of Lausanne approved the study and informed consent was obtained from all participants.

### Selection of SNPs, genotyping, and quality control procedure

In the discovery phase, all the participants in Radiant were genotyped using the Illumina HumanHap610-Quad BeadChips (Illuminia, Inc., San Diego, CA, USA) by the Centre National de Génotypage as previously described [24]. All DNA samples underwent stringent quality control including exclusion if the sample genotype missing rate was >1%, or if abnormal heterozygosity or unmatched sex assignment were observed. SNPs with minor allele frequency <1% or showing departure from the Hardy-Weinberg equilibrium (*P* <1 × 10^−5^) were excluded. Quality control was described in detail elsewhere [24]. The risk alleles were defined as alleles associated with increased risk of BMI. We derived a 32-SNP additive GRS from the SNPs reported by Speliotes et al. [9] and Belsky et al. [25]. Of the 32 GRS SNPs, 14 were extracted from GWAS data after applying quality control, and 13 were extracted using proxy SNPs with r^2^ > 0.9. The remaining 5 SNPs, namely rs11847697, rs11083779, rs11165643, rs7640855, and rs1475219, were derived from the 1000 Genomes project imputed data. The quality measure of imputation for these SNPs was above 0.8. The call rate for most SNPs was more than 96% except for one SNP, rs1475219, which was approximately 91%. The detailed information of the 32 SNPs is shown in Table 1.

**Table 1:** **Single nucleotide polymorphisms included in the genetic risk score in the RADIANT study**

**Chr**	**Nearest gene**	**SNP name**	**Alleles**	**BMI-increasing allele**	**Frequency of BMI-increasing allele**	**GWAS effect-size for BMI**	**Call rate**
1	NEGR1	rs2568958	A/G	A	62.5%	0.13	99.95%
	TNNI3K	rs1514175	A/G	A	42.3%	0.07	99.86%
	PTBP2	rs11165643	C/T	T	58.8%	0.06	99.07%
	SEC16B	rs10913469	C/T	C	19.2%	0.22	100%
2	TMEM18	rs2867125	C/T	C	82.9%	0.31	99.98%
	ADCY3,RBJ	rs10182181	A/G	G	46.9%	0.14	99.40%
	FANCL	rs759250	A/G	A	28.4%	0.1	100%
	LRP1B	rs6714473	C/T	T	9.7%	0.09	99.85%
3	CADM2	rs7640855	A/G	A	19.0%	0.1	96.83%
	ETV5	rs7647305	C/T	C	79.0%	0.14	99.93%
4	GNPDA2	rs12641981	C/T	T	44.1%	0.18	100%
	SLC39A8	rs13107325	C/T	T	7.5%	0.19	99.91%
5	FLJ35779	rs253414	C/T	T	66.4%	0.1	99.93%
	ZNF608	rs6864049	A/G	A	47.2%	0.07	100%
6	TFAP2B	rs987237	A/G	A	18.2%	0.13	100%
	NUDT3	rs206936	A/G	G	18.0%	0.06	95.99%
9	LRRN6C	rs2183825	C/T	C	32.9%	0.11	99.98%
11	STK33, RPL27A	rs10840065	A/G	A	51.6%	0.06	100%
	BDNF	rs6265	C/T	C	79.8%	0.19	100%
	MTCH2	rs10838738	A/G	G	34.5%	0.06	100%
12	BCDIN3, FAIM2	rs7138803	A/G	A	37.5%	0.12	100%
13	MTIF3	rs1475219	C/T	C	20.4%	0.09	90.61%
14	PRKD1	rs11847697	C/T	T	3.6%	0.17	96.87%
	NRXN3	rs10146997	A/G	G	21.9%	0.13	100%
15	MAP2K5	rs2241423	A/G	G	77.2%	0.13	99.96%
16	GPRC5B	rs12446632	A/G	G	86.1%	0.17	99.93%
	SH2B1	rs4788102	A/G	A	39.0%	0.15	100%
	FTO	rs3751812	G/T	T	41.0%	0.39	100%
18	MC4R	rs921971	C/T	C	26.6%	0.23	99.98%
19	KCTD15	rs29941	A/G	G	68.3%	0.06	100%
	ZC3H4, TMEM160	rs2303108	C/T	C	71.4%	0.09	100%
	QPCTL	rs11083779	C/T	T	95.8%	0.15	98.28%

The GSK Munich study was used for replication. Genotyping was performed using the Illumina HumanHap550 SNP Chip arrays. All SNPs with a call frequency below 95% were excluded. The details were described elsewhere [26]. The same criteria to construct the GRSs was applied here; whenever possible, SNPs were extracted from the GWAS data after applying quality control, and the rest of the SNPs were extracted using proxy SNPs.

Participants in the PsyCoLaus study were genotyped using the Affymetrix 500 K SNP chip [22]. The genotype was obtained via the BRLMM algorithm. The SNPs were removed from the analysis based on gender inconsistency, call rate less than 90%, and inconsistent duplicate genotypes. The GRSs were constructed as in the discovery phase.

### Construction of the unweighted and weighted GRS

To evaluate the combined effects of the 32 SNPs on BMI, an additive model was used to construct both unweighted and weighted GRSs. The unweighted GRS (uGRS) was calculated by summation of the number of risk alleles across the 32 variants. The weighted GRS (wGRS) was calculated by multiplying the number of risk alleles at each locus (0, 1, 2) for the corresponding effect sizes, in kg/m^2^ per allele, as reported by Speliotes et al. [9] and then summing the products. In order to reduce the bias caused by missing data, only the participants without any missing data were included in our GRS analysis.

### Statistical analysis

Linear regression models using traditional risk factors (age, sex, and principal components of ancestry) and GRS were calculated to predict BMI. Since BMI did not follow a normal distribution, a natural log-transformed BMI was used for the analyses. The analyses were first performed in the whole sample and then separately in the depressive cases and controls.

Binary logistic regression adjusted by age, sex, depression status and ancestry was used to predict probabilities of obesity in each model. Receiver-operating characteristics (ROC) curve analysis was conducted to calculate the area under the curve (AUC) to evaluate the discriminatory ability of each model. We first compared the difference between AUCs from models incorporating traditional risk factors (age, sex, and ancestry) with and without GRS. Then we compared the models comprising GRS only and the models incorporating other risk factors. To correct for the possible presence of population stratification, all analyses were adjusted for the first five principal components of ancestry, which were calculated with EIGENSOFT [27].

The analyses were performed first in the whole sample, and then separately in depressed patients and controls. All data were analyzed using STATA version 12.1 (STATA Corp, Texas). Two-tailed value of *P* <0.05 were considered significant.

## Results

### Discovery phase – Radiant study

#### Demographic characteristics

After excluding people with any missing genotypes, a total of 2,521 participants (2,086 non-obese and 435 obese) were included in the analysis. There were no differences in sex, age, and depression status between included and excluded people (all *P* >0.05). The mean age ± SD of participants was 43.9 ± 12.8 years (non-obese 43.2 ± 13.1, obese 47.3 ± 10.7, *t* = −6.08, *P* <0.0001) and 67.7% were female (72.9% female in obese and 66.6% female in non-obese, χ^2^ = 6.50, *P =* 0.011). Obese people were more likely to be depressed (90.3% vs. 72.0%, χ^2^ = 64.87, *P* <0.001).

**Figure 1:**
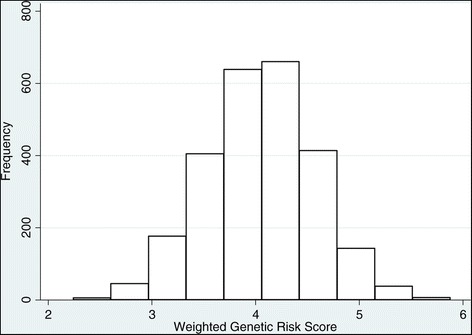
Distribution of weighted genetic risk score in RADIANT study.

The frequencies of uGRS and wGRS were approximately within normal distribution (Figure 1). The mean uGRS, the total number of risk alleles of 32 SNPs, was 29.5 ± 3.5 in obese and 28.6 ± 3.5 in non-obese participants (*t* = −4.47, *P* <0.0001), whereas the mean wGRS was slightly higher in obese compared to non-obese participants (4.14 ± 0.50 vs. 4.03 ± 0.53, *t* = −4.18, *P* <0.0001).

Principal component analysis was used to control for population stratification. The top five principal component scores were used to discriminate the subpopulation of white Europeans. Principal component 1 (distinguishes southeast Europe from northwest European ancestry) and principal component 2 (distinguishes east Europe from west Europe) were significantly associated with BMI and were included as covariates.

#### Linear regression analyses with BMI as the outcome variable

A base linear regression model including age, sex, depression status, ancestry, and significant interaction between ancestry and age accounted for 8.29% of the variance in log-transformed BMI. After adding weighted GRS to the base model, there was improvement of fit and an additional 1.27% of phenotypic variance of BMI explained giving a total of 9.56% (Table 2). Using either weighted or unweighted GRS made little difference for the explained variance of BMI (9.56% vs. 9.58%). No interaction between traditional covariates or between GRS and traditional covariates were found (data not shown). Although the interaction between depression and GRS on BMI did not meet the conventional 5% level of significance (ß = 0.27, s.e. = 0.02, *P* = 0.078), stratifying by depression status with GRS incorporated in the model explained an extra 1.63% of variance of BMI in depressed patients but only explained an extra 0.34% of variance of BMI in healthy controls.

**Table 2:** **Linear regression models with BMI as the outcome variable**

**Study/sample**	**Model**	**F**	**Adj. R** ^**2**^	**Additional variance explained by GRS**
***Radiant***				
Total	Model 1: adjusted by age, sex, and depression	38.98	0.0829	1.27%
	Model 2: model 1 + wGRS	39.16	0.0956	
Depressed cases	Model 1: adjusted by age and sex	17.85	0.0426	1.63%
	Model 2: model 1 + wGRS	20.75	0.0589	
Controls	Model 1: adjusted by age and sex	11.71	0.0789	0.34%
	Model 2: model 1 + wGRS	10.34	0.0823	
***GSK-Munich***				
Total	Model 1: adjusted by age, sex, and depression	34.02	0.1056	0.53%
	Model 2: model 1 + wGRS	29.80	0.1109	
Depressed cases	Model 1: adjusted by age and sex	8.02	0.0372	1.32%
	Model 2: model 1 + wGRS	7.13	0.0504	
Controls	Model 1: adjusted by age and sex	25.66	0.1306	0.23%
	Model 2: model 1 + wGRS	21.98	0.1329	
***PsyCoLaus***				
Total	Model 1: adjusted by age, sex, and depression	40.20	0.0843	0.93%
	Model 2: model 1 + wGRS	39.47	0.0936	
Depressed cases	Model 1: adjusted by age and sex	14.84	0.0605	1.09%
	Model 2: model 1 + wGRS	15.15	0.0714	
Controls	Model 1: adjusted by age and sex	31.25	0.0970	0.77%
	Model 2: model 1 + wGRS	29.21	0.1047	

#### Prediction of obesity

**Figure 2:**
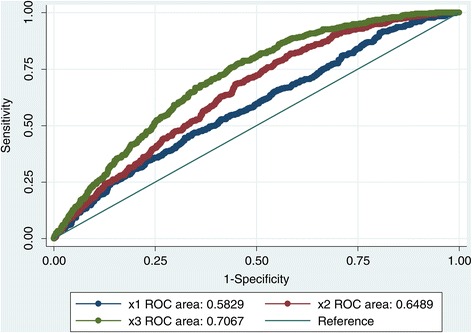
Receiver operating characteristic curves for models predicting obesity in the discovery phase. The AUC for the full model combining depression status, age, sex, and GRS (×3) is significantly greater than AUC for the model combining age, sex, and GRS (×2), which in turn is significantly greater than AUC for the base model with only GRS (×1).

Logistic regression models were used to examine the relationship between GRS and obesity in addition to age, sex, ancestry, and depression status. The discriminative power of the regression model was measured by the AUC. The AUC was significantly higher in the model combining all non-genetic risk factors (age, sex, ancestry, and depression status) and genetic factors compared to the model only applying non-genetic risk factors (AUC increased from 0.69 to 0.71, χ^2^ = 9.83, *P* = 0.0017). We further investigated whether GRS alone is able to discriminate obesity or not. The AUC was only 0.58 (95% CI, 0.55–0.61) while only including genetic risk score and ancestry into the base regression model. However, the AUC increased to 0.65 (95% CI, 0.62–0.68) after adding traditional risk factors such as age and sex (χ^2^ = 21.46, *P* <0.0001). The AUC further increased to 0.71 (95% CI, 0.68–0.73) on incorporating depression status into the above model (χ^2^ = 32.33, *P* <0.0001; Figure 2). Again, the unweighted GRS produced similar results as the wGRS when incorporated into our regression model (AUC increased from 0.58 to 0.65 to 0.70).

We used the same analysis stratifying by depression status and found that, in depressed patients, the AUC increased from 0.58 (95% CI, 0.55–0.61) to 0.61 (95% CI, 0.58–0.64; χ^2^ = 5.65, *P* = 0.0175) while in healthy controls it remained at 0.67 (95% CI, 0.60–0.73; χ^2^ = 0.00, *P* = 0.98). No interaction was found between depression, GRS, and obesity (OR = 1.08, s.e. = 0.36, *P* = 0.81).

### Replication phase – GSK Munich study

#### Demographic characteristics

A total of 1,679 participants (244 obese and 1,435 non-obese) were included in this study. The mean age ± SD was 51.49 ± 13.50 years (53.29 ± 11.51 for obese and 51.19 ± 13.80 for non-obese, *P* = 0.01). There was no sex difference between obese and non-obese patients (64.75% obese and 67.24% non-obese patients were female, *P* = 0.44). Obese people were more likely to be depressed (64.75% vs. 46.27%, *P* <0.001).

#### Linear regression analyses with BMI as the outcome variable

Linear regression models to predict BMI suggested the wGRS accounts for 0.63% of the variance in log-transformed BMI. While stratifying by depression status, we found wGRS explained an extra 1.32% of phenotypic variance of BMI in depressed patients but only accounted for 0.23% of variance in healthy controls (Table 2).

No significant interaction was found between depression and GRS on BMI (ß = 0.25, s.e. = 0.01, *P* = 0.18).

#### Prediction of obesity

Logistic regression models were used to examine the relationship between GRS and obesity in addition to age, sex, ancestry, and depression status. The AUC was approximately 0.59 (95% CI, 0.55–0.63) while only including genetic risk score and ancestry into the base regression model. The AUC increased to 0.64 (95% CI, 0.60–0.68) while adding traditional risk factors such as age and sex (χ^2^ = 8.21, *P* = 0.004). The AUC further increased to 0.69 (95% CI, 0.66–0.73) while incorporating depression status into the above model (χ^2^ = 10.67, *P* = 0.001). Stratified analyses by depression status showed that using wGRS to discriminate obesity was statistically significant in depressed patients (AUC increased from 0.53 (95% CI, 0.48–0.58) to 0.58 (95% CI, 0.53–0.63), χ^2^ = 4.19, *P* = 0.041) but not in healthy controls (AUC remained at 0.66 (95% CI, 0.60–0.72), χ^2^ = 0.34, *P* = 0.56).

No significant interaction was found between depression and GRS on obesity (OR = 1.38, s.e. = 0.39, *P* = 0.26).

### PsyCoLaus study

#### Demographic characteristics

Overall, 2,993 subjects (409 obese and 2,584 non-obese) were included in PsyCoLaus study. The mean age ± SD was 50.19 ± 8.84 years (52.94 ± 8.80 for obese and 49.76 ± 8.77 for non-obese, *P* <0.0001). There were no sex differences between obese and non-obese patients (49.87% of obese and 53.44% of non-obese people were female, *P* = 0.18). Obese people and non-obese people had equal depression rates (40.83% vs. 43.69%, *P* = 0.28).

#### Linear regression analyses with BMI as the outcome variable

Linear regression analysis to predict BMI suggested the wGRS accounts for 0.90% of the variance in log-transformed BMI. While stratifying by depression status, we found that wGRS explained an extra 1.09% of phenotypic variance of BMI in depressed patients but only accounted for 0.77% of variance of BMI in healthy controls (Table 2).

No significant interaction was found between depression and GRS on BMI (ß = 0.09, s.e. = 0.01, *P* = 0.52).

#### Prediction of obesity

Again, logistic regression models were used to examine the relationship between GRS and obesity in addition to age, sex, ancestry, and depression status. The AUC was approximately 0.56 (95% CI, 0.53–0.58) while only including GRS and ancestry into the base regression model. The AUC increased to 0.62 (95% CI, 0.59–0.65) while adding traditional risk factors such as age and sex (χ^2^ = 14.61, *P* = 0.0001). The AUC remained at 0.62 (95% CI, 0.59–0.65) while incorporating depression status into the above model (χ^2^ = 0.11, *P* = 0.74). Stratified analyses by depression status showed that using wGRS to discriminate obesity was not statistically significant neither in depressed patients (AUC increased from 0.61 (95% CI, 0.56–0.66) to 0.63 (95% CI, 0.58–0.67), χ^2^ = 3.66, *P* = 0.0558) nor in healthy controls (AUC increased from 0.61 (95% CI, 0.57–0.65) to 0.62 (95% CI, 0.59–0.66), χ^2^ = 2.66, *P* = 0.1).

No significant interaction was found between depression and GRS on obesity (OR = 0.98, s.e. = 0.21, *P* = 0.94).

## Discussion

In this study, we developed both weighted and unweighted GRS, including 32 well-established risk loci from a recent meta-analysis of GWAS on BMI [9]. We aimed to investigate whether these GRSs are associated with BMI and predict obesity.

### Prediction of BMI

Both uGRS and wGRS were associated with BMI (*P* <0.0001) and accounted for 1.27%, 0.63%, and 0.90% of phenotypic variance of BMI in Radiant, GSK Munich, and PsyCoLaus studies, respectively, and there was little difference in explained variance of BMI in each study. For each unit increase in uGRS, which is equal to one additional risk allele, BMI increased by approximately 0.175 kg/m^2^. Our overall result was thus in keeping with a previous study [9] using the same method to construct a GRS for BMI, but which did not take into account the relationship between BMI and depression.

Our results suggest that GRS explained more phenotypic variance of BMI in depressed patients than in healthy controls, although the interaction analyses were suggestive (Radiant) but not significant (GSK Munich and PsyCoLaus), this could reflect the fact that conventional levels of significance for interaction are often difficult to detect when the outcome variable has been log transformed. Interestingly, the case/control difference in the effect of GRS was more prominent when depression was diagnosed in clinical settings (RADIANT and GSK Munich studies) than in a community study (PsyCoLaus study).

### Prediction of obesity

We further explored the utility of a GRS approach using ROC analysis to compare the discriminatory ability of predictors of obesity. Conventionally, it is accepted that the AUC in a ROC analysis should be >0.8 to be of clinical value for screening. During the discovery phase, AUC fell short of this threshold but combining genetic factors and non-genetic factors proved better than using GRS alone in the prediction of obesity (with the AUC increasing from 0.69 to 0.71). In the replication phase, findings were similar except that depression had a small and non-significant association with obesity in the PsyCoLaus study, which could reflect the fact that PsyCoLaus was a community-based study with less severe cases of MDD than the clinically ascertained RADIANT and Munich GSK studies. Our results suggest that GRS might improve obesity prediction in depressed patients compared to controls.

In other respects, the results were similar to previous studies, which used only genome wide significant genetic variants to construct a GRS [11], in finding that the optimum AUC was obtained by combining GRS and non-genetic risk factors. A significant novel feature of the present study was that combining these factors with depression status further improves the prediction of obesity. This is in keeping with the association between obesity and MDD that has been found in either the general population or clinical settings [4,5,28]. Although the relationship between these two diseases may be bi-directional [5], our own recent analyses using a Mendelian Randomization approach [29] do not support a direction of cause from high BMI to depression. In addition, the fact that GRS has a larger effect on BMI and obesity in depressed patients, especially clinically severe depression, might reflect the importance of genetic effects on the association between obesity and clinically significant depression.

### Limitations

There are certainly some limitations that should be mentioned. First, we only selected the risk loci that reached genome-wide levels of significance. It is highly probable that there are additional as yet to be identified loci that will emerge when even larger sample sizes are included in GWAS. Second, since the established common variants from GWAS explain only a small proportion of the variation in BMI, future studies should include rare variants with larger effects and copy number variants to construct future GRS. In addition, gene-gene interactions and gene-environment interactions should be taken into account as well to maximize the obesity prediction ability of GRS. For example, our group [10] has found that depression status moderates the effect of *FTO* gene on BMI (although we did not find evidence of interaction between depression and GRS in the current study). Third, the 32 BMI loci used to construct the GRS were identified in GWAS of white European origin. The allele frequencies and their effect size may be different from non-European populations and the results should probably not be generalized to other ethnicities. Furthermore, the present study is a cross sectional study and cannot therefore take into account BMI fluctuations across the life span.

A further minor drawback is that PsyCoLaus is a subset of the CoLaus study, which was one of the 46 studies from which the GRS was derived [9], and therefore cannot, on its own, provide independent estimation of the risk score effect.

## Conclusions

In summary, we found that either a wGRS or a uGRS based on 32 well-established risk loci were significantly associated with BMI. Although GRS on its own explained only a small amount of variance of BMI, a significant novel feature of this study is that including non-genetic risk factors together with GRS and depression came close to the conventional threshold for clinical utility used in ROC analysis and improves the prediction of obesity.

Our results suggest that the GRS might predict obesity better in depressed patients than in healthy controls. This has potential clinical implications as well as implications for future research directions in exploring the links between depression and obesity-associated disorders.

While it is likely that future genome-wide studies with very large samples will detect variants other than the common ones, it seems probable that a combination of non-genetic information will still be needed to optimize the prediction of obesity.

## Additional file

Additional file 1:
**List of institutions where the ethical committees gave approval for the Radiant study.**

## Abbreviations

AUC: Area under the curve
BMI: Body mass index
DeCC: Depression case–control study
DeNT: Depression network study
*FTO*: Fat mass and obesity associated gene
GENDEP: Genome-based therapeutic drugs for depression
GRS: Genetic risk score
GWAS: Genome-wide association studies
MDD: Major depressive disorder
ROC: Receiver operating characteristic
SCAN: Schedules for Clinical Assessment in Neuropsychiatry
SNP: Single nucleotide polymorphism
uGRS: Unweighted genetic risk score
wGRS: Weighted genetic risk score
