# Supplementary material for: A genetic risk score combining 32 SNPs is associated with body mass index and improves obesity prediction in people with major depressive disorder
Source: BMC Med. 2015 Apr 17;13:86. doi: 10.1186/s12916-015-0334-3 (PMC4407390; doi:10.1186/s12916-015-0334-3)
Supplement: Additional file 1: — List of institutions where the ethical committees gave approval for the Radiant study. [file 12916_2015_334_MOESM1_ESM.docx]

**List of the names of institutions where the ethical committees gave approval for the Radiant study**

- Institute of Psychiatry, King’s College London, London, UK

- Max-Planck-Institute of Psychiatry, Munich, Germany

- Cardiff University School of Medicine, Cardiff, UK

-Trinity Centre for Health Sciences, Dublin, Ireland

- Barts and The London School of Medicine and Dentistry, Queen Mary's University of London, UK

- University of Bonn, Bonn, Germany.

- Aarhus University Hospital, Risskov, Denmark

- Lausanne University Hospital, Prilly-Lausanne, Switzerland

- Washington University School of Medicine, St Louis, MO, USA

- Central Institute of Mental Health, Mannheim, Germany

- Poznan University of Medical Sciences, Poland

- Université Libre de Bruxelles and – Belgium

- Medical School, University of Zagreb, Croatia
